# Desflurane inhalation before ischemia increases ischemia–reperfusion-induced vascular leakage in isolated rabbit lungs

**DOI:** 10.1186/s40064-016-3741-9

**Published:** 2016-11-29

**Authors:** Yoshiaki Oshima, Seiji Sakamoto, Kazumasa Yamasaki, Shinsuke Mochida, Kazumi Funaki, Naoki Moriyama, Akihiro Otsuki, Ryo Endo, Masato Nakasone, Shunsaku Takahashi, Tomomi Harada, Yukari Minami, Yoshimi Inagaki

**Affiliations:** Division of Anesthesiology and Critical Care Medicine, Department of Surgery, Tottori University Faculty of Medicine, 36-1 Nishi-cho, Yonago, Tottori 683-8504 Japan

**Keywords:** Desflurane, Ischemia–reperfusion injury, Isolated rabbit lungs, Coefficient of filtration

## Abstract

**Background:**

Isoflurane and sevoflurane protect lungs with ischemia–reperfusion (IR) injury. We examined the influence of desflurane on IR lung injury using isolated rabbit lungs perfused with a physiological salt solution.

**Methods:**

The isolated lungs were divided into three groups: IR, desflurane-treated ischemia–reperfusion (DES-IR), and ventilation/perfusion-continued control (Cont) groups (n = 6 per group). In the DES-IR group, inhalation of desflurane at 1 minimum alveolar concentration (MAC) was conducted in a stable 30-min phase. In the IR and DES-IR groups, ventilation/perfusion was stopped for 75 min after the stable phase. Subsequently, they were resumed. Each lung was placed on a balance, and weighed. Weight changes were measured serially throughout this experiment. The coefficient of filtration (K_fc_) was determined immediately before ischemia and 60 min after reperfusion. Furthermore, bronchoalveolar lavage fluid (BALF) was collected from the right bronchus at the completion of the experiment. After the completion of the experiment, the left lung was dried, and the lung wet-to-dry weight ratio (W/D) was calculated.

**Results:**

The K_fc_ values at 60 min after perfusion were 0.40 ± 0.13 ml/min/mmHg/100 g in the DES-IR group, 0.26 ± 0.07 ml/min/mmHg/100 g in the IR group, and 0.22 ± 0.08 (mean ± SD) ml/mmHg/100 g in the Cont group. In the DES-IR group, the K_fc_ at 60 min after the start of reperfusion was significantly higher than in the other groups. In the DES-IR group, W/D was significantly higher than in the Cont group. In the DES-IR group, the BALF concentrations of nitric oxide metabolites were significantly higher than in the other groups. In the DES-IR group, the total amount of vascular endothelial growth factor in BALF was significantly higher than in the Cont group.

**Conclusions:**

The pre-inhalation of desflurane at 1 MAC exacerbates pulmonary IR injury in isolated/perfused rabbit lungs.

## Background

Many studies reported that volatile anesthetics play a protective role against myocardial ischemia–reperfusion injury. The cardioprotective actions of these anesthetics are stated in the “ACC/AHA Guidelines for Perioperative Cardiovascular Assessment and Management after Non-cardiac Surgery” (Fleisher et al. 2007). Some articles have also reported the protective actions of volatile anesthetics against pulmonary ischemia–reperfusion injury (Liu et al. 1999, 2000).

Ischemia–reperfusion injury of the organs occurs through anoxia-reoxygenation. Anoxia rapidly decreases the level of adenosine triphosphate (ATP), leading to the generation of hypoxanthine. Reoxygenation promotes the production of reactive oxygen species, such as superoxide, hydrogen peroxide, and hydroxyradicals by hypoxanthine, inducing organ injury (de Perrot et al. 2003; Janero et al. 1991; Ambrosio et al. 1991).

In the lungs, the alveoli contain oxygen; therefore, oxygen deficiency does not occur rapidly. In the cardiac muscle, ATP level decreases to 50% within 6 min after circulatory arrest (Kolocassides et al. 1995). For lung tissue inflated with room air, 1.5 h are required until the ATP level decreases to 50% despite circulatory arrest (De Leyn et al. 1993). The pathogenesis of pulmonary ischemia–reperfusion injury differs from that of cardiac ischemia–reperfusion injury. Briefly, pulmonary blood flow disruption stimulates mechanoreceptors of the pulmonary vascular endothelial cells, causing lung injury through lipid peroxidation in these cells (de Perrot et al. 2003).

Lung tissue is more complex than cardiac muscle, consisting of the vascular endothelium and alveolar epithelium (de Perrot et al. 2003; Egan 2004). Pulmonary ischemia–reperfusion injury refers to damage of the pulmonary vascular endothelium and alveolar epithelium. Alveolar macrophages activated in the presence of ischemia–reperfusion release inflammatory cytokines (de Perrot et al. 2003). The influence of volatile anesthetics on pulmonary and cardiac ischemia–reperfusion injury may differ.

In a rat experimental model of neurogenic pulmonary edema, isoflurane exacerbated pulmonary edema by inducing the release of vascular endothelial growth factor (VEGF) in the vascular endothelium (Kandatsu et al. 2005); neurogenic pulmonary edema was caused by sympathetic excitement. Desflurane has a chemical formula similar to that of isoflurane (Eger 1987), but it has more potent stimulatory effects on the sympathetic nerve than isoflurane (Weiskopf et al. 1994). In addition, the blocked thoracic aorta causes oxidative stress in the entire organism through ischemia and subsequently pulmonary vascular permeability, which is exacerbated by desflurane (Nielsen et al. 1998). In addition, in pigs under mechanical ventilation, desflurane (1 minimum alveolar concentration [MAC]) inhalation caused oxidative stress in the lungs and induced the apoptosis of lung cells (Allaouchiche et al. 2001; Kalimeris et al. 2011). In the present study, we hypothesized that desflurane could exacerbate increased vascular permeability in ischemic reperfusion injured lungs through sympathetic stimulatory effects, and examined this hypothesis using isolated perfused rabbit lungs. Vascular hyperpermeability was evaluated based on the capillary coefficient of filtration (K_fc_).

## Methods

### Isolated perfused lung preparation

The experimental protocol was approved by the Tottori University Laboratory Animal Care Committee. Isolated rabbit lungs were prepared using the method described in detail by Liu et al. (1999) with minor modifications. Male New Zealand White rabbits weighing 1.9–2.6 kg were used. The lungs were ventilated with 93.5% air and 6.5% CO_2_ (tidal volume 6 ml/kg; frequency 40/min; positive end-expiratory pressure 2 cmH_2_O) and perfused with bicarbonate-buffered physiologic salt solution [PSS (in mM): NaCl, 119; KCl, 4.7; MgSO_4_, 1.17; NaHCO_3_, 22.61; KH_2_PO_4_, 1.18; CaCl_2_, 3.2] in a recirculating manner at a constant flow rate of 30 ml/kg/min. To each 100 ml of PSS stock solution, we added 100 mg dextrose, 20 mU insulin, and 5 g hydroxyethylstarch (Ajinomoto Pharmaceuticals, Tokyo, Japan). Pulmonary arterial pressure (P_PA_), left atrium pressure (P_LA_), and airway pressure (P_AW_) were monitored continuously via side ports in the respective cannula. The partial pressure of carbon dioxide (PCO_2_) in the perfusate was adjusted to 35–40 mmHg during the experiment by continuous aeration of the reservoir liquid surface using mixed gas with the same composition as the inspired gas. The empyreal part of the reservoir was covered with Saran Wrap^®^, and the liquid surface of the reservoir was separated from air. Gas analysis of the perfusate was performed during the experiment, and a small amount of sodium bicarbonate was added to the perfusate to maintain the pH at 7.35–7.45.

The lungs were removed en bloc and enclosed in a humidified chamber which was positioned on an electronic scale (GX4000; A and D, Tokyo, Japan) to allow continuous monitoring of lung weight. P_LA_ was set at 2 mmHg (referenced at the hilum), and the whole system was equilibrated at 37 °C. A flow probe (FF-050T; Nihon Kohden, Tokyo, Japan) connected to an electromagnetic flow meter (MFV-3100; Nihon Kohden, Tokyo, Japan) was placed in the perfusion circuit for the continuous monitoring of blood flow (Q). Total pulmonary vascular resistance (R_t_) was calculated using the following formula: R_t_ = (P_PA_ − P_LA_)/Q.

### Coefficient of filtration

Pulmonary vascular permeability was evaluated by determining the pulmonary capillary K_fc_ in accordance with the Starling equation (Drake et al. 1978). First, isogravimetric pressure (P_ISO_) was measured as described by Thompson et al. (1997): the shunt between the pulmonary and left atrial cannulae was opened and perfusion was discontinued, and changes in lung weight were observed by gradually increasing the reservoir height and pulmonary capillary pressure (P_PC_) to determine maximum P_PC_ without any increase in lung weight, i.e. P_ISO_. The P_PC_ was then changed to equal P_ISO_ +7 mmHg by rapidly elevating the reservoir to a height corresponding to +7 mmHg, and the reservoir was kept at this height for 7 min. The rate of lung weight gain every minute from 2 to 6 min was then recorded on a semilogarithmic plot and extrapolated to time 0 by linear regression. The logarithm of lung weight gain at time 0 was converted to an antilog, and the resulting value was used to calculate K_fc_.

K_fc_ was normalized using the baseline wet lung weight and expressed in mL/min/mmHg/100 g lung tissue. The baseline wet lung weight was calculated from the body weight (BW) of the animals using the formula: BW (g) × 0.0024 (Seeger et al. 1986).

Although positive pressure ventilation was interrupted during the K_fc_ determination, a constant flow of the mixed gas was administered at 3 cmH_2_O of P_AW_.

### Wet-to-dry weight ratio

The left lung was excised at the end of the experiment and its wet weight was measured. The left lung was dried at 85 °C under a heating lamp for 30 h and its dry weight was measured to determine lung water weight compared with pulmonary tissue weight to determine the wet-to-dry weight ratio (W/D) using the formula: W/D = (wet weight − dry weight)/dry weight.

### Bronchoalveolar lavage fluid analysis

The right lung was used for BALF preparation at the end of the experiment. Four aliquots (5 ml each) of isotonic sodium chloride solution were instilled separately through the trachea and drained. The collected fluid was centrifuged immediately at 250×*g* and 4 °C for 10 min. The supernatant was divided into several aliquots and stored at −80 °C until analysis.

The concentration of nitric oxide (NO) metabolites (sum of NO_3_^−^ and NO_2_^−^) was determined using a high-performance liquid chromatography system (Shimadzu, Tokyo, Japan) with visible light absorbance detection at 546 nm, as described by Green et al. (1982).

Lactate dehydrogenase (LDH) concentration was measured using a clinical chemistry analyzer (JCA-BM8060, Japan Electron Optics Laboratory, Tokyo, Japan). The lower detection limit was 6 IU/l.

Superoxide dismutase (SOD) activity was measured by the modified nitrite method, as described by Oyanagui (1984).

Interleukin (IL)-6 concentration was measured using a fully automated chemiluminescent enzyme immunoassay system (Lumipulse F, Fujirebio Inc., Tokyo, Japan). The lower detection limit was 0.2 pg/ml.

VEGF concentration was measured using an enzyme immune assay (EIA) kit (hVEGF QKit, R&D Systems Inc., Minneapolis, USA). As the concentration of VEGF in BALF reduces through a pulmonary edema fluid-related dilutional effect, we compared the total amount of VEGF in each BALF sample (Cross and Matthay 2011; Bhargava and Wendt 2012; Ware et al. 2005).

### Experimental protocol

The isolated lungs selected were those that (1) had a homogenous white appearance without signs of hemostasis or edema formation, and (2) were isogravimetric in the equilibration period of 30 min.

The isolated lungs were divided into three groups. In the control (Cont) group (*n* = 6), the isolated lungs were continuously perfused and ventilated after an equilibration period for 135 min. In the ischemia–reperfusion (IR) group (*n* = 6), ventilation and perfusion were interrupted (ischemia) after an equilibration period for 75 min, and the isolated lungs were maintained in the humidified chamber at 37 °C while P_AW_ was maintained at 3.5 cmH_2_O by administrating a constant flow of mixed gas. Gas at the same concentration and flow rate as used in the equilibration period was added to the liquid surface of the reservoir. The empyreal part of the reservoir was covered with Saran Wrap^®^, and the liquid surface of the reservoir was separated from the air. The pulmonary arterial and left atrial cannula were both opened to the atmosphere, resulting in an intravascular pressure of 0 mmHg during ischemia. The isolated lungs were reperfused and reventilated for 60 min after ischemia. The flow velocity at the start of reperfusion was increased slowly to 30 ml/kg/min, so that the P_PA_ did not exceed 20 mmHg. At the start of reventilation, the positive end-expiratory pressure was established as 10 cmH_2_O for a few respirations until atelectasis was macroscopically relieved, but the same respiratory conditions as adopted in the equilibration period were used thereafter. In the desflurane-treated ischemia–reperfusion (DES-IR) group (*n* = 6), 1 MAC [1 MAC = 8.9% for rabbits (Loer et al. 1995)] desflurane was administered for 30 min during the equilibration period, then ischemia was performed for 75 min, followed by 60 min reperfusion and reventilation, which was the same as that for the IR group.

In a preliminary experiment, the pre-inhalation of desflurane at 1.5 MAC was performed. At the start of reperfusion, edema fluid appeared in a tracheal tube before the flow rate reached 30 ml/kg/min, and it was impossible to continue the protocol. In this experiment, the inhalation concentration of desflurane was established as 1 MAC.

Desflurane (Baxter International Inc., Chicago, IL) was administered using a desflurane vaporizer (D-Vapor, Dräger Medical GmbH, Lübeck, Germany). The concentration of desflurane in inspired gas was monitored using a bedside monitor (BSM-2301, Nihon Kohden, Tokyo, Japan).

K_fc_ was determined in the IR and DES-IR groups at baseline (end of the equilibration period) and 60 min after the start of reperfusion. R_t_, P_AW_, and lung weight gain were determined in the IR and DES-IR groups at baseline and at 10, 20, 30, 40, 50, and 60 min after the start of reperfusion. These values were determined in the Cont group at the same intervals. When edema fluid appeared in a tracheal tube before the flow velocity reached 30 ml/kg/min at the start of reperfusion, the K_fc_ and wet left lung weight were measured at that point, and BALF was collected from the right lung. The experiment was then completed.

Statistical analysis was performed using Prism 5 (Graph Pad Software, San Diego, CA). All data are presented as the mean ± standard deviation (SD). Changes in K_fc_, R_t_, lung weight gain, and P_aw_ were evaluated using two-way factorial analysis of variance (ANOVA) followed by the post hoc Bonferroni test. Changes in NO metabolites, LDH, SOD, IL6, and VEGF in the BALF and the W/D were evaluated using one-way factorial ANOVA or Kruskal–Wallis test followed by post hoc Tukey test or Dunn’s test. Significance was determined at P < 0.05.

## Results

In the DES-IR group, there was an isolated lung preparation in which edema fluid appeared in a tracheal tube before the flow velocity reached 30 ml/kg/min at the time of reperfusion. In this preparation, it was impossible to measure the R_t_, P_AW_, and lung weight gain after reperfusion.

### Coefficient of filtration

**Fig. 1:**
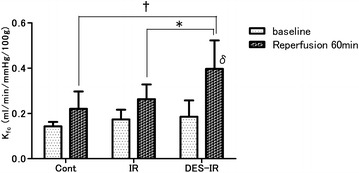
Changes in the coefficient of filtration (K_fc_). Data are mean ± SD (n = 6 per group). ^δ^P < 0.0001 versus baseline. *P < 0.05 versus DES-IR group. ^†^P < 0.01 versus DES-IR group

The K_fc_ values at 60 min after reperfusion were 0.40 ± 0.13 ml/min/mmHg/100 g in the DES-IR group, 0.26 ± 0.07 ml/min/mmHg/100 g in the IR group, and 0.22 ± 0.08 ml/min/mmHg/100 g in the Cont group. In the DES-IR group, the K_fc_ at 60 min after reperfusion was significantly higher than in the IR (P < 0.05) and Cont (P < 0.01) groups. It was significantly higher than the baseline (P < 0.0001) (Fig. 1).

### Wet-to-dry weight ratio

**Fig. 2:**
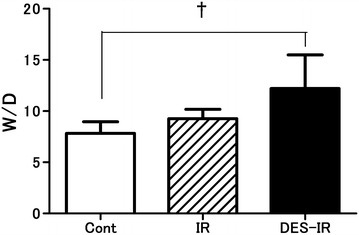
Changes in lung wet-to-dry weight ratio (W/D). Data are mean ± SD (n = 6 per group). ^†^P < 0.01 versus DES-IR group

The W/D ratios were 12.2 ± 3.3 in the DES-IR group, 9.3 ± 0.9 in the IR group, and 7.8 ± 1.1 in the Cont group. In the DES-IR group, the W/D was significantly higher than in the Cont group (P < 0.01) (Fig. 2).

### Physiological parameters analysis

The DES-IR group consisted of 5 isolated lung preparations, and the other two groups consisted of 6 each.

**Fig. 3:**
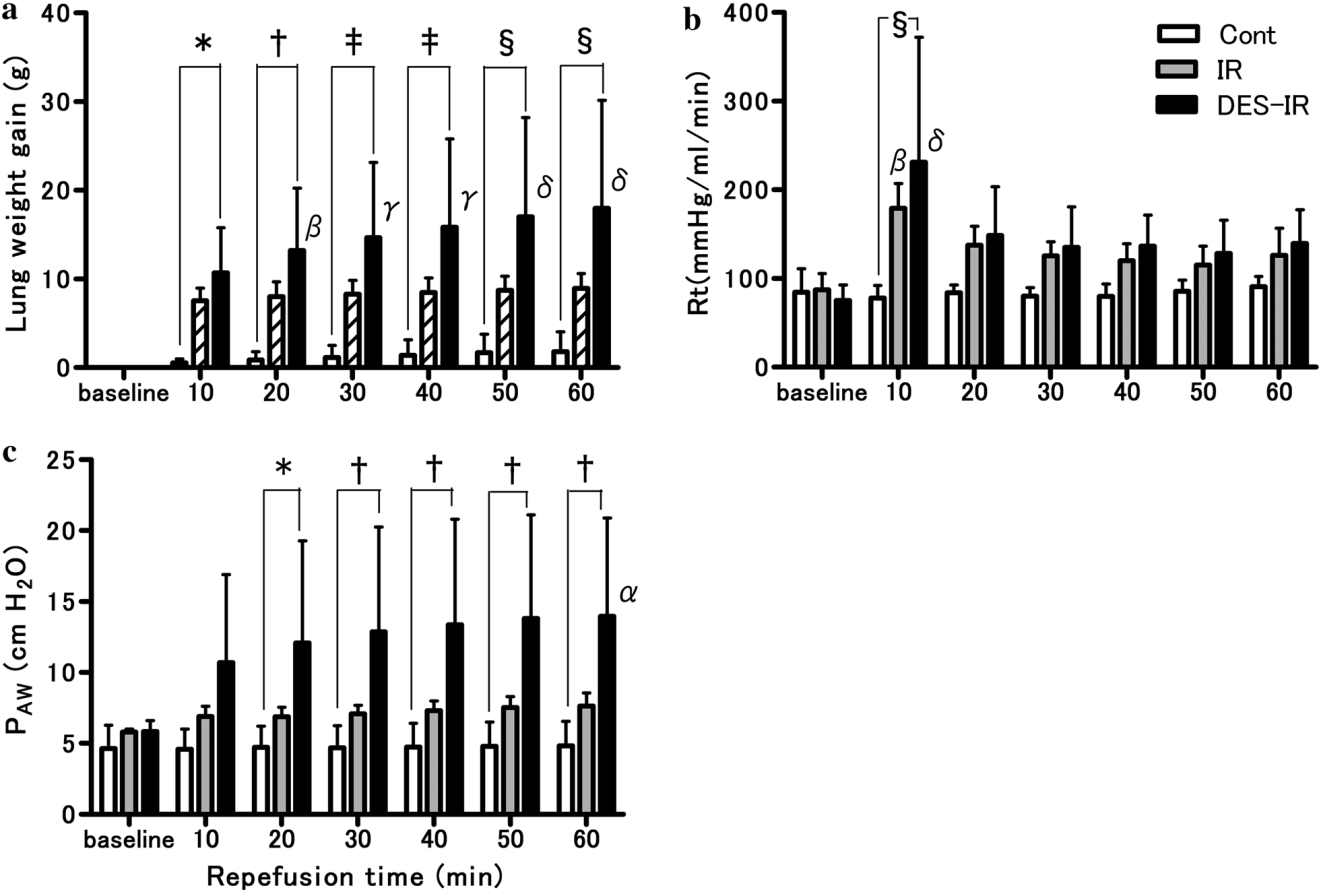
Physiological parameters after reperfusion (n = 6 per group). One of six experiments in the DES-IR group was terminated because of severe lung edema within 10 min after the start of reperfusion. Therefore, the data from this animal were removed from the analysis. Data are mean ± SD. **a** Lung weight gain. **b** R_t_: total pulmonary vascular resistance. **c** P_AW_: airway pressure. *P < 0.05 versus Cont group. ^†^P < 0.01 versus Cont group. ^‡^P < 0.001 versus Cont group. ^§^P < 0.0001 versus Cont group. ^α^P < 0.05 versus baseline. ^β^P < 0.01 versus baseline. ^γ^P < 0.001 versus baseline. ^δ^P < 0.0001 versus baseline

In the DES-IR group, the lung weight gain at 10, 20, 30, 40, 50, and 60 min after the start of reperfusion was significantly higher than in the Cont group (P < 0.05, P < 0.01, P < 0.001, P < 0.001, P < 0.0001, and P < 0.0001, respectively). In this group, the lung weight gain at 20, 30, 40, 50, and 60 min after the start of reperfusion was significantly higher than the baseline (P < 0.01, P < 0.001, P < 0.001, P < 0.0001, and P < 0.0001, respectively) (Fig. 3a).

In the DES-IR group, the R_t_ at 10 min after reperfusion was significantly higher than in the Cont group (P < 0.0001). In the DES-IR and IR groups, there were significant increases in R_t_ 10 min after reperfusion in comparison with the baseline (P < 0.0001, and P < 0.01, respectively) (Fig. 3b).

In the DES-IR group, the P_AW_ at 20, 30, 40, 50, and 60 min after the start of reperfusion was significantly higher than in the Cont group (P < 0.05, P < 0.01, P < 0.01, P < 0.01, and P < 0.01, respectively). In this group, the P_AW_ at 60 min after the start of reperfusion was significantly higher than the baseline (P < 0.05) (Fig. 3c).

### Bronchoalveolar lavage fluid analysis

**Fig. 4:**
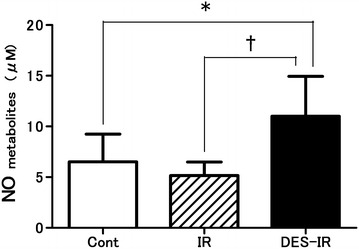
Changes in nitric oxide (NO) metabolite (sum of nitrite and nitrate) concentrations in bronchoalveolar lavage fluid (BALF). Data are mean ± SD (n = 6 per group). *P < 0.05 versus DES-IR group. ^†^P < 0.01 versus DES-IR group

The NO metabolite concentrations in BALF were 11.0 ± 4.0 μM in the DES-IR group, 5.2 ± 1.3 μM in the IR group, and 6.5 ± 2.7 μM in the Cont group. In the DES-IR group, the BALF concentrations of NO metabolites were significantly higher than in the Cont and IR groups (P < 0.05, and P < 0.01, respectively) (Fig. 4).

**Fig. 5:**
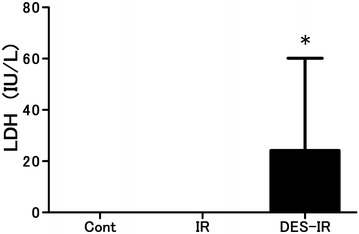
Changes in lactate dehydrogenase (LDH) concentration in bronchoalveolar lavage fluid (BALF). (LDH concentrations in all samples of BALF from the Cont and IR groups were lower than the detection level). Data are mean ± SD (n = 6 per group). *P < 0.05 versus other groups

In the DES-IR group, the BALF concentration of LDH was significantly higher than in the other groups (P < 0.05). In all samples of BALF belonging to the Cont and IR groups, the concentrations of LDH were below the detection limit (Fig. 5).

**Fig. 6:**
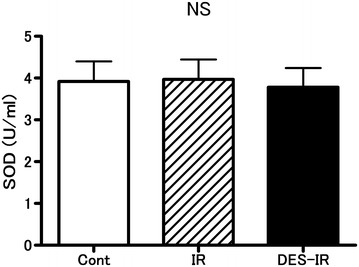
Changes in superoxide dismutase (SOD) activity in bronchoalveolar lavage fluid (BALF) (n = 6 per group). Data are mean ± SD. There were no significant differences among the three groups (*NS* not significant)

The SOD activity in BALF was 3.8 ± 1.1 U/ml in the DES-IR group, 4.0 ± 1.2 U/ml in the IR group, and 3.9 ± 1.2 U/ml in the Cont group. There were no significant differences in SOD activity in BALF among the three groups (Fig. 6).

**Fig. 7:**
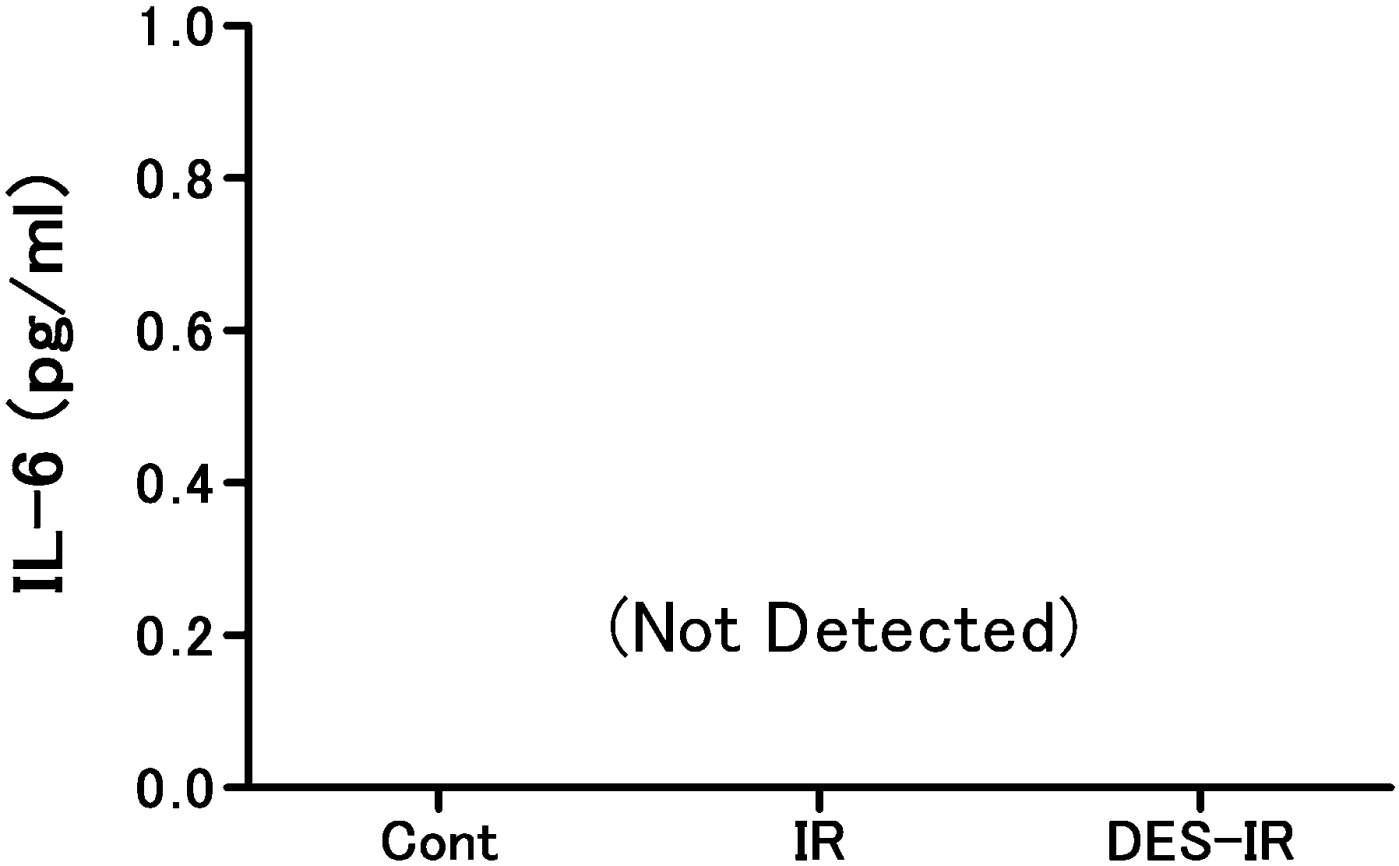
Changes in interleukin (IL)-6 concentration in bronchoalveolar lavage fluid (BALF). Data are mean ± SD (n = 6 per group). IL-6 concentrations in all samples of BALF from the three groups were less than detection level

In all samples of BALF belonging to the three groups, the BALF concentrations of IL-6 were below the detection limit (Fig. 7).

**Fig. 8:**
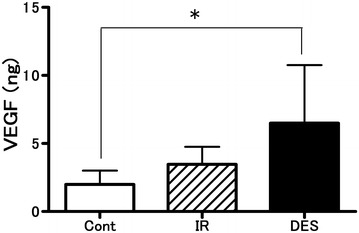
Changes in total amount of vascular endothelium growth factor (VEGF) in bronchoalveolar lavage fluid (BALF). Data are mean ± SD (n = 6 per group). *P < 0.05 versus DES-IR group

The total amounts of VEGF in BALF were 6.5 ± 4.3 ng in the DES-IR group, 3.5 ± 1.3 ng in the IR group, and 2.0 ± 1.0 ng in the Cont group. In the DES-IR group, the total amount of VEGF in BALF was significantly higher than in the Cont group (P < 0.05). It was slightly higher than in the IR group, although there was no statistical difference (Fig. 8).

## Discussion

Using isolated/perfused rabbit lungs, we examined the influence of desflurane on lungs with ischemia–reperfusion injury. In the DES-IR group, K_fc_ at 60 min after the start of reperfusion was significantly higher than the baseline. It was also significantly higher than in the other groups. In addition, W/D in the DES-IR group at the completion of this experiment was significantly higher than in the Cont group. Furthermore, the BALF concentrations of NO metabolites were also significantly higher than in the other groups. In the DES-IR group, the total amount of VEGF in BALF was significantly higher than in the Cont group.

The mechanism of neurogenic pulmonary edema involves the marked enhancement of the sympathetic nerves. Kandatsu et al. (2005) reported that pre-treatment inhalation of 1.5% isoflurane exacerbated neurogenic pulmonary edema in a rat in vivo neurogenic pulmonary edema model. This was because isoflurane stimulated VEGF release by pulmonary vascular endothelial cells. The vascular permeability-enhancing action of VEGF was 50,000 times stronger than that of histamine (Dvorak et al. 1995). VEGF receptors are also present in the alveolar epithelium (Maniscalco et al. 1997).

An in vivo experiment using hairless albino guinea pigs demonstrated that the vascular permeability-enhancing action mechanism of VEGF involved the VEGF-induced release of NO and prostacyclin in the vascular endothelium (Murohara et al. 1998). Another experiment using cultured vascular endothelial cells from the bovine microcirculatory system also showed that the vascular endothelial cells produced NO and prostacyclin in the presence of VEGF stimulation (Murohara et al. 1998).

Desflurane is a halogenated methyl ethyl ether, similar to isoflurane. The structural formulae of desflurane and isoflurane differ in the halogen atom bound to the α carbon atom of the ethyl group, which chlorine in isoflurane and fluorine in desflurane (Eger 1987). Isoflurane and desflurane have common characteristics, such as concentration-dependent sympathomimetic effects. In healthy adult volunteers, the sympathomimetic effects of desflurane were more marked than those of isoflurane (Weiskopf et al. 1994).

In this experiment, in the DES-IR group, vascular resistance after reperfusion was high, and the total weight of VEGF in BALF was significantly higher than in the Cont group, and slightly higher than in the IR group. In addition, the concentrations of NO metabolites in BALF were higher than in the other groups. In addition, the pre-inhalation of desflurane potentiated ischemia–reperfusion injury-related pulmonary vascular hyperpermeability. This was possibly because desflurane induced the release of VEGF by pulmonary vascular endothelial cells through a mechanism similar to that of isoflurane.

We used the release of LDH into the BALF as an estimate of cell damage during the experiment. At the completion of this experiment, the BALF concentration of LDH in the DES-IR group was higher than in the other groups. This indicated not only the decreased functions of the vascular endothelium, but also damage to lung tissue (Nielsen et al. 1998).

Kandatsu et al. (2005) reported that the pre-inhalation of 1.5% isoflurane exacerbated neurogenic pulmonary edema by inducing the release of VEGF in vascular endothelial cells in a rat in vivo neurogenic pulmonary edema model, whereas pre-inhalation of 2.5% sevoflurane reduced pulmonary edema without inducing VEGF release. In a study using perfused rat lungs, the pre-inhalation of isoflurane at 1 MAC protected against pulmonary vascular hyperpermeability after warm ischemia–reperfusion, but the protective effects of isoflurane at 3 MAC were weak (Fujinaga et al. 2006). This was possibly because isoflurane at 3 MAC inhibited cyclic guanosine monophosphate production, increasing vascular resistance at 10 min after ischemia–reperfusion. According to another study, in pigs under mechanical ventilation, the inhalation of desflurane at 1 MAC increased the concentration of malondialdehyde (MDA) in BALF, whereas sevoflurane at 1 MAC did not increase it (Allaouchiche et al. 2001). Volatile inhalation anesthetics have both damaging and protective effects on the lungs depending on their types and inhalation concentrations.

Several methods can be employed to quantify the microvascular damage caused by ischemia–reperfusion injury. The most reliable is a histological examination of the lungs, which allows a detailed examination of the region and extent of damage. However, this method does not provide sufficient quantitative data on the functional status of the microvascular barrier. In contrast, K_fc_, measured in isogravimetric conditions, can be reliably and reproducibly determined in the isolated and perfused lungs of dogs, rabbits, rats, and guinea pigs. K_fc_ has been measured in isolated and perfused lungs in studies on various reperfusion injuries and microvessels in other lung injuries (Liu et al. 2000; Thompson et al. 1997). A high K_fc_ value reflects high capillary permeability. In the present study, K_fc_ also corresponded to W/D.

The present study had some limitations, as described below. In this experiment using perfused rabbit lungs, the levels of IL-6 in BALF in the three groups were below the detection limit. Kotani et al. reported that volatile inhalation anesthetics increased the BALF levels of tumor necrosis factor-α and macrophage inflammatory protein 2, as an inflammatory cytokine, in rats on mechanical ventilation, whereas the BALF level of IL-6 was below the detection limit, showing no change (Kotani et al. 1999). Chung et al. (2013) indicated that the homogenized lung tissue level of IL-6 increased using a rat lipopolysaccharide-induced lung injury model. IL-6 is released by macrophages, lymphocytes, vascular endothelial cells, fibroblasts, and mast cells (Liu et al. 2007). It may have been necessary to measure the lung tissue level of IL-6 in this experiment. In our experiment, the SOD activity in BALF remained unchanged even after desflurane inhalation. In an in vivo experiment with rabbits, desflurane (1 MAC) inhalation enhanced pulmonary vascular permeability by oxidants released by the transient blocking of the thoracic aorta and reduced the ascorbic acid content in lung tissue (Nielsen et al. 1998). Ascorbic acid is a biological antioxidant. In pigs under artificial respiration, desflurane (1 MAC) inhalation resulted in oxidative stress in the lungs, induced the apoptosis of lung cells, and increased the MDA concentration in BALF, but caused no change in SOD activity (Allaouchiche et al. 2001; Kalimeris et al. 2011). In this experiment with rabbits, MDA concentrations in BALF should have been measured. In addition, Liu et al. (2000) demonstrated the protective effects of sevoflurane on ischemia–reperfusion injuries of isolated perfused lungs of rats, but not of rabbits. Thus, in the present study, the same experiment should have been conducted with sevoflurane. In addition, to further demonstrate that VEGF-stimulated NO release increased vascular permeability, the effects of desflurane on the vascular permeability of ischemia–reperfusion injured lungs pretreated with NO-inhibitors, such as L-NAME, should have been examined. Furthermore, histological changes in the ischemia–reperfusion injured lungs, caused by desflurane, should have been examined.

Among volatile anesthetics, desflurane shows the most potent protective actions on cardiac muscle (Piriou et al. 2002). In cardiac surgery using cardiopulmonary bypass (CPB), desflurane may be used for myocardial protection. However, CPB poses a risk of pulmonary ischemia–reperfusion injury. Thus, the use of desflurane at a high concentration in cardiac surgery using CPB may cause lung injuries.

## Conclusions

We investigated the influence of desflurane on the lungs with ischemia–reperfusion injury using isolated/perfused rabbit lungs. The pre-inhalation of desflurane exacerbated ischemia–reperfusion-induced pulmonary vascular hyperpermeability and pulmonary damage.

## Abbreviations

MAC: minimum alveolar concentration
K_fc_: coefficient of filtration
BALF: bronchoalveolar lavage fluid
W/D: lung wet-to-dry weight ratio
NO: nitric oxide
VEGF: vascular endothelial growth factor
ATP: adenosine triphosphate
P_PA_: pulmonary arterial pressure
P_LA_: left atrium pressure
P_AW_: airway pressure
PCO_2_: partial pressure of carbon dioxide
Q: blood flow
R_t_: total pulmonary vascular resistance
P_ISO_: isogravimetric pressure
P_PC_: pulmonary capillary pressure
BW: body weight
LDH: lactate dehydrogenase
SOD: superoxide dismutase
IL: interleukin
EIA: enzyme immune assay
SD: standard deviation
ANOVA: analysis of variance
MDA: malondialdehyde
CPB: cardiopulmonary bypass
